# Ultrasensitive detection of 2,4-dichlorophenoxyacetic acid by inhibiting alkaline phosphatase immobilized onto a highly porous gold nanocoral electrode†

**DOI:** 10.1039/d4nr04857a

**Published:** 2025-03-25

**Authors:** Angelo Tricase, Michele Catacchio, Verdiana Marchianò, Eleonora Macchia, Paolo Bollella, Luisa Torsi

**Affiliations:** a Department of Pharmacy-Pharmaceutical Sciences, University of Bari Aldo Moro Via E. Orabona 4-70125 Bari Italy; b Centre for Colloid and Surface Science, University of Bari Aldo Moro Via E. Orabona 4-70125 Bari Italy paolo.bollella@uniba.it; c Faculty of Science and Engineering, Åbo Akademi University 20500 Turku Finland; d Department of Chemistry, University of Bari Aldo Moro Via E. Orabona 4-70125 Bari Italy

## Abstract

Herein, we describe the design and implementation of an ultrasensitive enzyme inhibition-based biosensor for 2,4-dichlorophenoxyacetic acid (2,4-D) detection. The biosensor utilizes alkaline phosphatase (AlP), immobilized on a photo-crosslinked polymer matrix of poly(vinyl alcohol) functionalized with *N*-methyl-4(4′-formylstyryl)pyridinium (PVA-SbQ), supported by electrodes coated with highly porous gold nanocorals (hPGNCs). After preliminary electrochemical and morphological characterization, the PVA-SbQ/AlP/hPGNC electrode was tested for inhibition studies employing ascorbate 2-phosphate (A2P) as the initial substrate. The biosensor preparation/sensing time from electrode preparation to final results is approximately 45 minutes, which enables the possibility to easily scale up the electrode production process on a daily basis with a reliable analytical result in only 5 minutes of amperometric measurement. Following the initial kinetic studies and evaluation of analytical performance, the PVA-SbQ/AlP/hPGNC platform demonstrated a linear detection range from 0.002 to 22 ppt, with a sensitivity of 0.121 ± 0.006 ppt^−1^ (RSD = 4.9%, *R*^2^ = 0.996, and *N* = 6) and a limit of detection (LoD) of 0.7 ppq. This sensitivity is 7–8 orders of magnitude below the regulatory thresholds in Europe and the USA. Furthermore, the biosensor was validated using 19 homogenized wheat leaf sample extracts, prepared in line with European Food Safety Authority (EFSA) guidelines, achieving average recoveries exceeding 96% and RSD values under 9.8%. The biosensor also exhibited robust operational and storage stability, maintaining 84% (30 hours of continuous operation) and 94% (120 days) of its initial response, respectively. These results highlight the potential of the PVA-SbQ/AlP/hPGNC biosensor for on-site 2,4-D monitoring in agricultural crops and its feasibility for integration with artificial intelligence for advanced diagnostics.

## Abstract 2

Herein, we describe the design and implementation of an ultrasensitive enzyme inhibition-based biosensor for 2,4-dichlorophenoxyacetic acid (2,4-D) detection.

## Graphical abstract

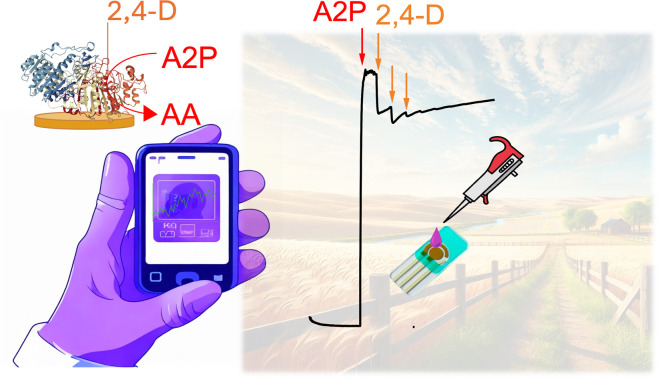

## 1. Introduction

2,4-Dichlorophenoxyacetic acid (2,4-D) is an herbicide acting as a growth regulator, primarily used to control broadleaf weeds in cereal crops.^1–3^ The presence of 2,4-D residues in food and the environment presents serious health risks to humans and animals.^4–6^ To reduce these risks, the US Environmental Protection Agency (EPA) has set a maximum contaminant level (MCL) for 2,4-D at 0.07 mg L^−1^ (70 ppb).^7,8^ Based on the European Regulation (EC) no 1107/2009, further revised in the Commission Implementing Regulation (EU) 2015/2033, the Theoretical Maximum Daily Intake (TMDI) of 2,4-D for all considered consumer groups is estimated to be 3.2% of the Acceptable Daily Intake (ADI, 0.02 mg per kg body weight per day), namely 0.64 ppb.^9,10^ Therefore, developing an effective and quick method for detecting 2,4-D is vital for ensuring the safety of food and the environment.^11,12^

Several traditional analytical techniques have been used to detect 2,4-D and other environmental contaminants.^13,14^ While these methods offer high sensitivity, selectivity, and specificity, they exhibit several limitations, including the need for expensive equipment, complex sample processing, and specialized expertise.^15,16^ Additionally, these conventional approaches are not ideal for on-site pollutant monitoring. Notably, there has been growing focus on developing alternative analytical tools that minimize sample preparation, reduce costs, shorten analysis time, and enable on-site measurements.^17–19^

Biosensors are increasingly recognized for their potential in environmental monitoring. Several enzyme- and antibody-based biosensors have been created for detecting 2,4-D, using various transducer technologies (*e.g.*, electrochemical, surface plasmon resonance (SPR), optical methods, *etc.*).^20–23^ Despite their effectiveness, these biosensors often face challenges, including the instability of antibodies and enzymes under certain conditions. More recently, electrochemical sensors using molecularly imprinted polymers (MIPs) have shown significant promise, as the imprinted materials offer improved mechanical and chemical stability.^24–26^ Furthermore, biosensors employing whole cells, like *Pseudomonas putida* and *Anabaena torulosa*, have been developed for detecting 2,4-D.^27^ Enzyme inhibition-based biosensors are particularly suitable for *in situ* and continuous environmental analysis.^28–33^ These biosensors allow the study of the kinetics of the inhibition process, which requires modifications to conventional theories and equations to account for heterogeneous phases. It is also important to recognize that many biosensor signals represent transient and/or local equilibria, which may not align with traditional enzyme-based models, and enable the quantification of inhibitor concentrations by assessing the percentage of inhibition on the immobilized biocatalysts.^34,35^

Over the past two decades, numerous studies have focused on developing inhibition-based biosensors for detecting environmental pollutants.^36,37^ For instance, several AlP-based biosensors have been developed for the detection of 2,4-D by measuring the inhibition of AlP activity, providing a rapid and sensitive detection method.^24,25^ Additionally, a biosensor combining AlP with glucose oxidase has been reported for 2,4-D detection, enhancing sensitivity through a dual-enzyme approach.^38^ Another study has developed a biosensor for detecting both 2,4-D and malathion using AlP and substrates like 3-indoxyl phosphate, phenyl phosphate, or ascorbate-2-phosphate, demonstrating versatility in detecting multiple pollutants.^39–41^ Furthermore, Sok *et al.* explored the use of carbon nano-onion peroxidase composite biosensors for the electrochemical detection of 2,4-D and 2,4,5-trichlorophenoxyacetic acid (2,4,5-T), highlighting advancements in nanomaterial-based biosensors.^42^

**Fig. 1:**
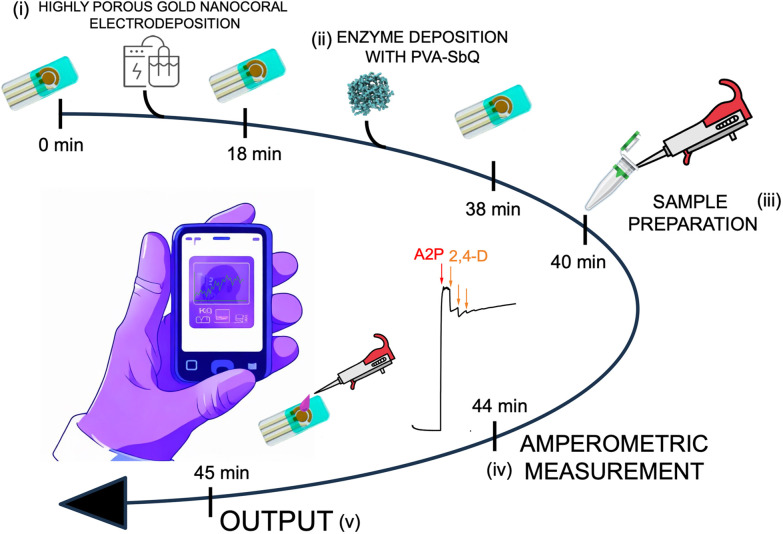
Schematic representation of the preparation/sensing time: (i) highly porous gold nanocoral electrodeposition (hPGNC), (ii) alkaline phosphatase (AlP) immobilization with UV-Vis at *λ* = 405 nm in the presence of *N*-methyl-4(4′-formylstyryl)pyridinium (PVA-SbQ) as a photo-crosslinked polymer, (iii) sample preparation encompassing the addition of ascorbate 2-phosphate (A2P) as an initial substrate, (iv) amperometric measurement of enzyme inhibition, and (v) final output.

This study reports for the first time the development of an ultrasensitive enzyme inhibition-based biosensor for 2,4-D detection, where alkaline phosphatase (AlP) was entrapped through a photo-crosslinked polymer, namely poly(vinyl alcohol), *N*-methyl-4(4′-formylstyryl)pyridinium (PVA-SbQ), onto highly porous gold nanocoral (hPGNC) modified electrodes. After preliminary electrochemical and morphological characterization, the PVA-SbQ/AlP/hPGNC electrode was tested for inhibition studies employing ascorbate 2-phosphate (A2P) as the initial substrate. As reported in Fig. 1, the biosensor preparation/sensing time from electrode preparation to final results is approximately 45 minutes, which enables the possibility to easily scale up the electrode production process on a daily basis with a reliable analytical result in only 5 minutes of amperometric measurement. Finally, the biosensor was applied to detect 2,4-D in spiked wheat leaf extract samples.

## 2. Results and discussion

### 2.1 Electrochemical and morphological characterization of a highly porous gold nanocoral (hPGNC) electrode

**Fig. 2:**
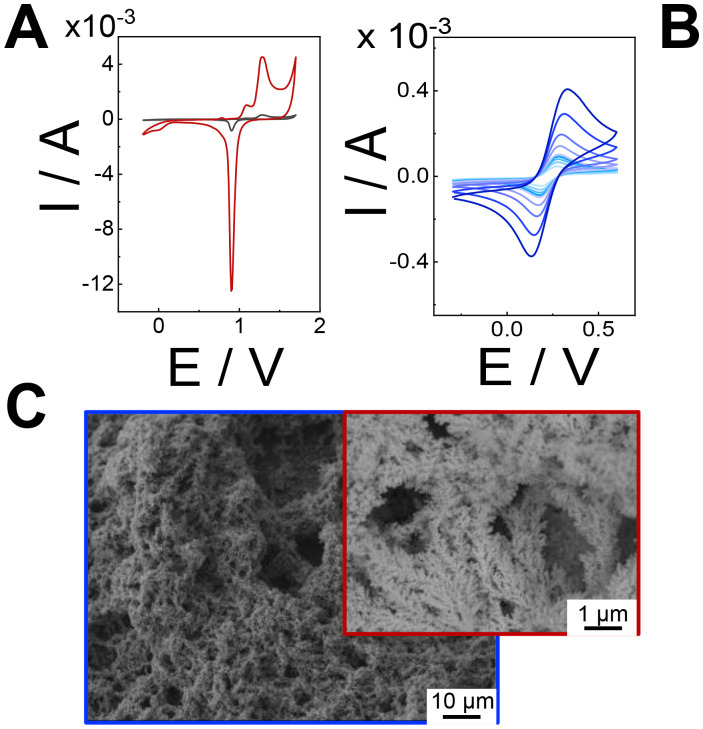
(A) CVs of the bare gold screen printed electrode (black) and the hPGNC modified electrode (red) in 0.5 M H_2_SO_4_. Scan rate: 100 mV s^−1^. (B) CVs of the hPGNC modified electrode in 5 mM [Fe(CN)_6_]^3−/4−^ in 10 mM HEPES buffer pH 7 (containing 100 mM Na_2_SO_4_ as a supporting electrolyte) at increasing scan rates (colour coded) in the range 5–300 mV s^−1^. (C) SEM images of the hPGNC modified electrode.

To demonstrate the improvements of our electrodeposition method, the real electroactive area (*A*_real_) was determined by integrating the peak current associated with the reduction of gold oxide (approximately +0.9 V) during cyclic voltammetry (CV) in 0.5 M H_2_SO_4_ at a scan rate of 100 mV s^−1^, as shown in Fig. 2A. The theoretical charge density for the reduction of gold oxide was considered to be 390 ± 10 μC cm^−2^.^43^ As a result, the *A*_real_ value of the hPGNC modified electrodes was 9.58 ± 0.63 cm^2^ (Fig. 2A, red curve), which is approximately 96 times greater than that of the bare screen-printed gold electrodes (0.1 ± 0.02 cm^2^, Fig. 2A black curve). The roughness factor (*ρ*) could be estimated as 76.3 ± 5.1 with respect to the geometric area of the screen-printed gold electrode (notably 0.1256 cm^2^). Additionally, hPGNC modified electrodes were analysed at varying scan rates (Fig. 2B) to calculate the electroactive area (*A*_ea_), roughness factor (*ρ*), and electron transfer rate constant (*k*^0^, cm s^−1^).^44^ The hPGNC modified electrodes exhibited an *A*_ea_ value of 16.2 ± 1.2 cm^2^, a roughness factor of 128.9 ± 9.5 (derived by dividing the electroactive area by the geometric area), and an electron transfer rate constant of (3.2 ± 0.2) × 10^−2^ cm s^−1^. The electroactive area was calculated using the Randles–Ševčík equation.^45^ The electron transfer rate constants (*k*^0^, cm s^−1^) were estimated through a combined approach incorporating the Klingler–Kochi method and the Nicholson and Shain method for both irreversible and reversible systems.^46^ Similar to other highly porous nanostructured electrode surfaces, *A*_ea_ was higher than *A*_real_ due to the complex nanostructuration occurring at the edge of the highly porous structure. The structure porosity correlates also with the rate of hydrogen bubble production during the pulsed electrodeposition steps, affecting the morphology of the electrode surface.^20,47^

SEM analysis was conducted to examine the morphology of the hPGNC substrates. As shown in Fig. 2C, the surfaces coated with hPGNC reveal a multimodal pore size distribution, with defined pores having diameters ranging from 1 to 5 μm, not homogeneously distributed onto the whole electrode surface. Moreover, the fine pore structure revealed the presence of coral-like nanostructures, hereinafter defined as nanocorals, with an average size of 50–200 nm.

hPGNC electrodes have been characterized through XPS analysis at each electrodeposition step: only cyclic voltammetry (CV, sample 1), cyclic voltammetry and pulsed amperometry (CV + pA, sample 2) and only pA (sample 3), as reported in Fig. S1 (ESI†). The high-resolution spectra of the gold bare electrode and hPGNC show characteristic peaks for the Au 4f signal. The expected Au 4f_7/2_ binding energy (BE) is 83.8 ± 0.5 eV, which was observed for the bare gold electrode and CV modified electrode (sample 1), indicating minimal surface changes.^48^ However, a slight increase in the gold content and a decrease in the carbon and oxygen contents were noted (Table S1†). Significant changes occur in CV + pCA and pCA electrodes. These samples exhibit a noticeable shift in the gold signal due to pore formation, with the BE increasing to 84.7 ± 0.5 eV. The gold atomic percentage also rises significantly, reaching 59 ± 5% in sample 2 and 62 ± 5% in sample 3 (Table S1†), but the latter experiences greater surface degradation.

Valence Band (VB) analysis, particularly near the Fermi level, confirms this shift, which is attributed to the quantum confinement effect.^49^ The formation of nanocorals alters the electronic structure, affecting electron energy levels. The confined dimensions of nanocorals influence electron behavior, while interactions between surface atoms and metal electrons further modify their energy states.^50^

### 2.2 Kinetic characterization of the PVA-SbQ/AlP/hPGNC electrode

**Fig. 3:**
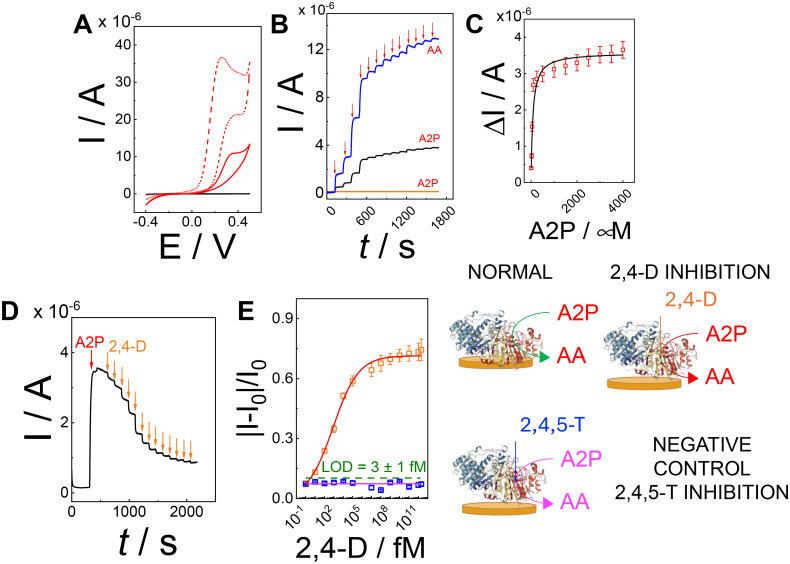
(A) CVs of the PVA-SbQ/AlP/hPGNC electrode in 10 mM HEPES buffer pH 7 (containing 1 mM MgSO_4_ as a cofactor and 100 mM Na_2_SO_4_ as a supporting electrolyte, black curve) with the addition of 2 mM AA (red dashed curve) and 2 mM A2P (red solid curve) recorded at 5 mV s^−1^. (B) The amperometric curve of the PVA-SbQ/AlP/hPGNC electrode recorded by applying *E* = +0.35 V and adding A2P in the concentration range 10–4000 μM every 100 s (black curve); the PVA-SbQ/hPGNC electrode recorded by applying *E* = +0.35 V and adding AA (blue curve) and A2P (orange curve) in the concentration range 10–4000 μM every 100 s. Addition of AA and A2P (red arrows). (C) Calibration plot of the PVA-SbQ/AlP/hPGNC electrode with Michaelis–Menten fitting (black curve) for data extracted from the amperometric curve recorded by applying *E* = +0.35 V and adding A2P in the concentration range 10–4000 μM. (D) The amperometric curve of the PVA-SbQ/AlP/hPGNC electrode recorded by applying *E* = +0.35 V and adding 2 mM A2P (red arrow) as a substrate, and 2,4-D in the concentration range 1 fM (1 × 10^−15^ M) to 2 mM (2 × 10^−3^ M) (orange arrows) as an inhibitor, every 100 s. (E) Calibration plot of the PVA-SbQ/AlP/hPGNC electrode with Hill fitting applied to the mixed inhibition model for data extracted from the amperometric curve of the PVA-SbQ/AlP/hPGNC electrode recorded in the presence of 2,4-D in the concentration range 1 fM (1 × 10^−15^ M) to 2 mM (2 × 10^−3^ M) (orange dots, red fitting line) and 2,4,5-T in the same concentration range as a negative control (blue dots and magenta fitting line); insets: schematic representation of the catalytic process and inhibition process occurring at the AlP modified electrode.

Afterwards, CV experiments were performed in 10 mM HEPES buffer pH 7 (containing 1 mM MgSO_4_ as a cofactor and 100 mM Na_2_SO_4_ as a supporting electrolyte) to evaluate the enzymatic efficiency in the catalytic conversion of A2P in ascorbic acid (AA). Fig. 3A reports the catalytic CV (red dashed curve) in the presence of 2 mM AA showing an onset potential (*E*_ONSET_) of −0.17 V (comparatively lower than that of the bare gold electrode, reported at 0 V) with a maximum current peak of 36.4 ± 2.7 at +0.22 V. Conversely, the catalytic CV (red solid curve) in the presence of 2 mM A2P shows an *E*_ONSET_ of +0.010 V with a maximum current peak of 11.2 ± 0.9 at +0.31 V. The shift in the current peak potential might be ascribed to the interfacial kinetics and diffusion limitations occurring at the enzyme/electrode interface during the conversion of A2P to AA.^51^

Fig. 3B reports the amperometric curve (black curve) recorded for the PVA-SbQ/AlP/hPGNC electrode by applying *E* = +0.35 V with the sequential addition of A2P in the concentration range 10–4000 μM (control experiments are reported for the PVA-SbQ/hPGNC electrode in the presence of AA (blue curve) and A2P (orange curve)). The amperometric curve was further fitted according to the Michaelis–Menten equation, as follows (eqn (1)):1
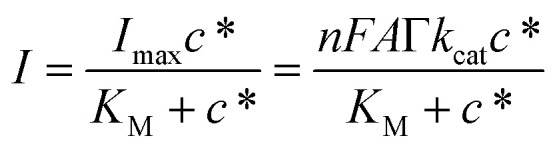
where *K*_M_ is the Michaelis–Menten constant, *k*_cat_ is the turnover constant for A2P conversion to AA, *Γ* is the enzymatic surface coverage (namely AlP), c* is the substrate concentration (namely A2P) and *I*_max_ is the maximum catalytic current, while *n*, *F* and *A* have their usual meaning. Fig. 3C depicts the fitted curve (black line) based on the data extracted from the amperometric output (Fig. 3B) represented as a scattered plot, resulting in a *K*_M_ value of 78.9 ± 3.6 μM and an *I*_max_ value of 3.6 ± 0.1 μA (*R*^2^ = 0.982). The *k*_cat_ was computed as 0.22 ± 0.01 s^−1^ (obtained considering an enzymatic surface coverage of 8.5 pmol cm^−2^ estimated through the p-NPP enzymatic assay) and a *k*_cat_/*K*_M_ value of 2788 s^−1^ M^−1^. All enzymatic parameters estimated through the amperometric data agree with other results reported in the literature.^39,40^

**Fig. 4:**
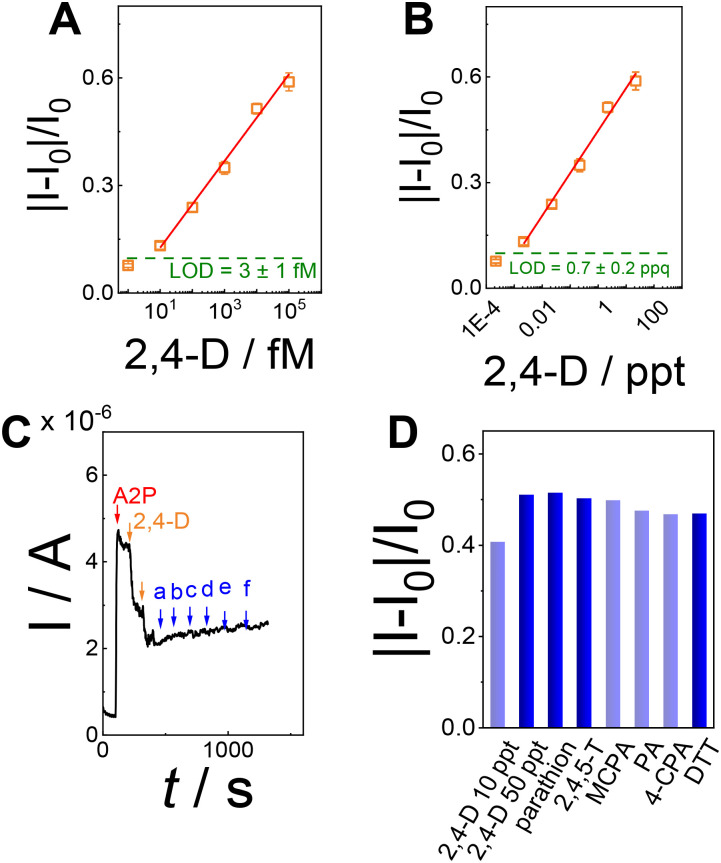
(A) Linear range extracted from the calibration plot for the PVA-SbQ/AlP/hPGNC electrode recorded in the presence of 2,4-D in the concentration range 1 fM (1 × 10^−15^ M) to 2 mM (2 × 10^−3^ M) (orange dots, red fitting line); the linear range between 10 fM (1 × 10^−15^ M) and 100 pM (100 × 10^−12^ M). (B) The linear range reported to be in the range 0.002–22 ppt of 2,4-D for the PVA-SbQ/AlP/hPGNC electrode. (C) The amperometric curve to test the interfering compounds by adding 10 and 50 ppt of 2,4-D (in the presence of A2P as a substrate) and testing sequentially (a) parathion, (b) 2,4,5-T, (c) MCPA, (d) PA, (e) 4-CPA and (f) DDT at a concentration of 100 ppt. Experimental parameters: *E* = +0.35 V; A2P 2 mM. (D) Bar diagram for amperometric responses of the PVA-SbQ/AlP/hPGNC electrode in the presence of (a) parathion, (b) 2,4,5-T, (c) MCPA, (d) PA, (e) 4-CPA and (f) DDT at a concentration of 100 ppt.

After preliminary enzymatic characterization, the PVA-SbQ/AlP/hPGNC electrode was further assessed by performing amperometric experiments in the presence of A2P as a substrate (indicated with red arrows) followed by the addition of sequential concentration of 2,4-dichlorophenoxyacetic acid (2,4-D, orange arrows) in the range 1 fM (1 × 10^−15^ M) to 2 mM (2 × 10^−3^ M) as an analytical target (acting as an inhibitor of the AlP enzymatic activity, Fig. 3D), and 2,4,5-trichlorophenoxyacetic acid (2,4,5-T) as a negative control. The calibration curves of PVA-SbQ/AlP/hPGNC for both 2,4-D (orange dots, red fitted line) and 2,4,5-T (blue dots, magenta fitted line) are reported in Fig. 3E and fitted with the Hill equation. Recently, AlP was reported to exhibit a mixed inhibition. In this model, the inhibitor can bind to both the enzyme–substrate complex and the free enzyme, but with different affinities, making the inhibition both competitive (affecting the substrate binding) and non-competitive (affecting the AlP activity). The Hill equation for mixed inhibition is an extension of the Michaelis–Menten equation that accounts for both competitive and non-competitive inhibition, with the added consideration of cooperativity.^25^

In the case of mixed inhibition, the inhibitor binds to both the free AlP and the AlP-A2P complex, but with different affinities. This can be described using the following equation (eqn (2)):2
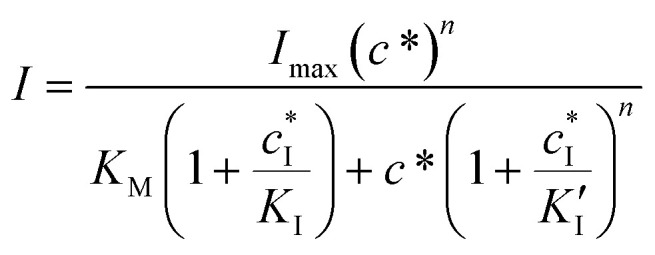
where 
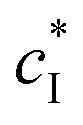
 is the inhibitor concentration, *K*_I_ is the inhibition constant for the free AlP (competitive inhibition part), 
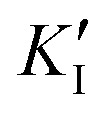
 is the inhibition constant for the AlP-A2P complex (non-competitive inhibition part), and *n* is the Hill coefficient (reflects cooperativity of the AlP–A2P interaction), while *K*_M_, *I*_max_, and c* have their usual meaning. Hence, *K*_I_ and 
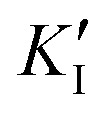
 are 827 ± 98 fM and 785 ± 63 fM, respectively, while the Hill coefficient (*n*) is 0.32 ± 0.01 (*R*^2^ = 0.997 and *N* = 6). In this case, the PVA-SbQ/AlP/hPGNC electrode exhibited negative cooperativity, where the binding of 2,4-D molecules reduced the affinity of AlP for additional A2P molecules. Similarly, the same fitting model was applied to 2,4,5-T inhibition showing no inhibiting response on AlP.

### 2.3 Analytical characterization of the PVA-SbQ/AlP/hPGNC electrode

Furthermore, the calibration curve reported in Fig. 3E reported a limit of detection (LoD) of 3 ± 1 fM calculated as 
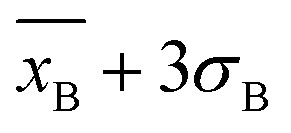
, considering the average current response reported for 2,4,5-T as a negative control. The calibration curve exhibited a linear range between 10 fM (1 × 10^−15^ M) and 100 pM (100 × 10^−12^ M), with a sensitivity of 0.121 ± 0.006 fM^−1^ (RSD = 4.9%, *R*^2^ = 0.996, and *N* = 6, Fig. 4A). Using the calibration curve, the LoD can be estimated as follows (eqn (3)):3
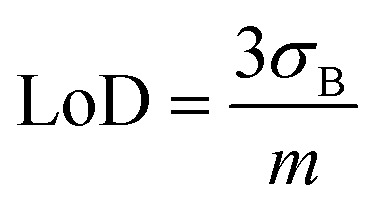
where *σ*_B_ is the standard deviation of the blank response (current measured for 2,4,5-T as a negative control) and *m* is the slope of the calibration curve. The calculated LoD for the PVA-SbQ/AlP/hPGNC electrode was 3.2 ± 1.1 fM. Since 2,4-D is a contaminant, the MRLs and threshold are reported according to parts-per notation. Hence, the linear range was calculated in the range 0.002–22 ppt, with a sensitivity of 0.121 ± 0.006 ppt^−1^ (RSD = 4.9%, *R*^2^ = 0.996, and *N* = 6) and a LoD of 0.7 ppq (Fig. 4B), which is 7–8 orders of magnitude lower than the law limit established in both Europe and the USA.^8,52^

The selectivity of PVA-SbQ/AlP/hPGNC was investigated by adding 1 and 10 ppt of 2,4-D (in the presence of A2P as a substrate) and testing sequentially 100 ppt of potentially inhibiting pesticides, as reported in Fig. 4C. The bar diagram (Fig. 4D) shows a 5–7% decrease in the response for parathion, 2,4,5-T and MCPA because organophosphate pesticides can act as AlP inhibitors, while for 2,4,5-T and MCPA, there is a minimal structural difference.^26^ Similarly, PA, 4-CPA and DDT show a decrease of 8–9% in the analytical signal, due to the structural similarity (most of the inhibiting function occurs at the level of the aromatic ring interacting with the AlP active site), while DDT is a well-known organochlorine pesticide, and similar to other organochlorine compounds, it has been shown to have toxic effects on various biological systems, including enzyme inhibition.^53^

**Fig. 5:**
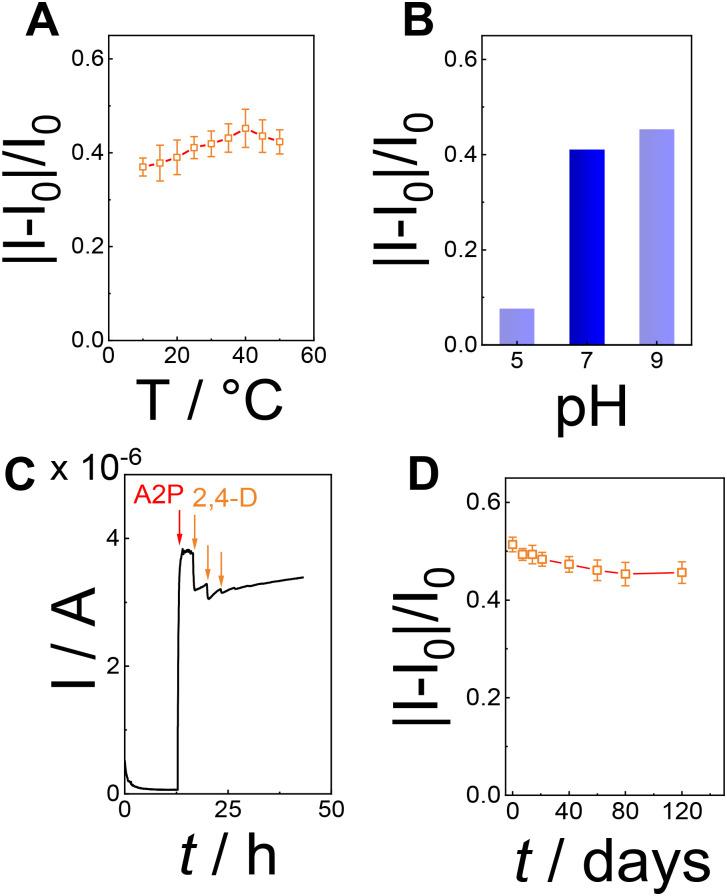
(A) Temperature effect measured for the PVA-SbQ/AlP/hPGNC electrode in the presence of 10 ppt 2,4-D. Experimental parameters: *E* = +0.35 V; A2P 2 mM. (B) Bar diagram of the pH effect measured for the PVA-SbQ/AlP/hPGNC electrode in the presence of 10 ppt 2,4-D. Experimental parameters: *E* = +0.35 V; A2P 2 mM. (C) Operational stability of the PVA-SbQ/AlP/hPGNC electrode measured over 30 hours of continuous measurements after the addition of 1 and 10 ppt 2,4-D; experimental parameters: *E* = +0.35 V; A2P 2 mM. (D) Storage stability of the PVA-SbQ/AlP/hPGNC electrode measured in the presence of 10 ppt 2,4-D over 120 days; experimental parameters: *E* = +0.35 V; A2P 2 mM.

The effects of temperature and pH are depicted in Fig. 5A and B, respectively. Regarding temperature dependence, the PVA-SbQ/AlP/hPGNC electrode demonstrated the best performance at 40 °C. However, the biosensor maintained good activity at both room temperature and 40 °C. Above 40 °C, the current density dropped, likely due to enzyme denaturation. The optimal pH was found to be 9 in a 10 mM Tris buffer, which aligns with other AlP-inhibition biosensors. However, the catalytic response is comparable also at pH 7 in a 10 mM HEPES buffer (decreasing by 12%), while the current output decreased sharply at acidic pH, which agrees with previous literature findings.^24,25^

The operational stability of PVA-SbQ/AlP/hPGNC was tested in the presence of A2P as a substrate followed by two sequential additions of 2,4-D up to 10 ppt, retaining 84% of the initial response over 30 hours of continuous measurement (Fig. 5C). Additionally, the storage stability of PVA-SbQ/AlP/hPGNC was evaluated considering the current response measured for 2,4-D inhibition at 10 ppt over 120 days, retaining 94% of the initial activity (Fig. 5D).

To validate the potential of the PVA-SbQ/AlP/hPGNC electrode for the on-site detection of 2,4-D as a screening tool for analytical process control in wheat farming, the proposed biosensor was employed to measure 2,4-D concentrations in 19 homogenized wheat leaf sample extracts collected and processed following the procedure outlined in the European Food Safety Authority (EFSA) guidelines.^54^ The real wheat samples, labelled (numerical order) and collected from different local farms, were spiked with 3 different concentrations of 2,4-D, namely 0.1, 1 and 10 ppt, as reported in Table 1.

**Table 1:** Results obtained for 2,4-D spiked wheat leaf sample extract analysed with the PVA-SbQ/AlP/hPGNC electrode (SD values are reported for each sample)

Sample ID	Found value/ppt	Nominal value/ppt	Recovery %
#1001	1.02 ± 0.11	1	102
#1002	9.74 ± 0.18	10	97.4
#1003	0.08 ± 0.01	0.1	80
#1004	0.92 ± 0.09	1	92
#1005	0.09 ± 0.01	0.1	90
#1006	0.08 ± 0.01	0.1	80
#1007	0.91 ± 0.13	1	91
#1008	0.88 ± 0.14	1	88
#1009	0.95 ± 0.11	1	95
#1010	10.42 ± 0.18	10	104.2
#1011	0.09 ± 0.01	0.1	90
#1012	10.11 ± 0.21	10	101
#1013	1.18 ± 0.18	1	118
#1014	0.11 ± 0.01	0.1	110
#1015	9.82 ± 0.21	10	98
#1016	1.12 ± 0.19	1	112
#1017	1.08 ± 0.25	1	108
#1018	0.09 ± 0.01	0.1	90
#1019	0.08 ± 0.01	0.1	80

The proposed biosensor demonstrates good performance across all homogenized wheat leaf samples, with average recovery values of 88.6% (RSD <11.4% calculated based on average recovery values) for 0.1 ppt, 100.8% (RSD <12.4% calculated based on average recovery values) for 1 ppt, 100.2% (RSD <3.5% calculated based on average recovery values) for 10 ppt 2,4-D spiked wheat sample extract, respectively. Moreover, the average recovery is above 96% with an RSD below 9.8%, proving the ability of the PVA-SbQ/AlP/hPGNC electrode to reliably measure 2,4-D in farming samples. The proposed biosensor PVA-SbQ/AlP/hPGNC was compared with other platforms reported in the literature reporting the lowest LoD, high sensitivity, and longer operational and storage stability (Table S2†).^28,55–58^

## 3. Conclusions

This study reports the development of an ultrasensitive enzyme inhibition-based biosensor for detecting 2,4-D, where alkaline phosphatase (AlP) is immobilized *via* a photo-crosslinked polymer, poly(vinyl alcohol)-*N*-methyl-4(4′-formylstyryl)pyridinium (PVA-SbQ), on electrodes modified with highly porous gold nanocorals (hPGNCs). After preliminary kinetics and analytical performance, the PVA-SbQ/AlP/hPGNC biosensing platform exhibited a linear range of 0.002–22 ppt, with a sensitivity of 0.121 ± 0.006 ppt^−1^ (RSD = 4.9%, *R*^2^ = 0.996 and *N* = 6) and a LoD of 0.7 ppq, which is 7–8 orders of magnitude lower than the law limit established in both Europe and the USA. Additionally, the PVA-SbQ/AlP/hPGNC electrode was tested in 19 homogenized wheat leaf sample extracts according to the European Food Safety Authority (EFSA) guidelines, reporting an average recovery above 96% with an RSD below 9.8%. The proposed biosensor also showed good operational and storage stability retaining 84% (30 hours of continuous operation) and 94% of the initial response (120 days), respectively. The overall analytical performance confirms the possibility of employing the PVA-SbQ/AlP/hPGNC biosensor for on-site 2,4-D analysis in farming crops and the possibility of being coupled with artificial intelligence as a diagnostic tool.

## Author contributions

A.T., M.C., and V.M. wrote a part of the manuscript. E.M., P.B. and L.T. supervised A.T. and M.C. on data collection and analysis. L.T., E.M. and P.B. conceived the experimental work, revised the manuscript and are responsible for funding acquisition. All authors approved the final version.

## Data availability

Data for this article are available at Zenodo at https://doi.org/10.5281/zenodo.14172625.

## Conflicts of interest

There are no conflicts to declare.

## Supplementary Material

NR-017-D4NR04857A-s001
