# Supplementary material for: Ultrasensitive detection of 2,4-dichlorophenoxyacetic acid by inhibiting alkaline phosphatase immobilized onto a highly porous gold nanocoral electrode
Source: Nanoscale. 2025 Mar 25;17(15):9197–204. doi: 10.1039/d4nr04857a (PMC11934013; doi:10.1039/d4nr04857a)
Supplement: NR-017-D4NR04857A-s001 [file NR-017-D4NR04857A-s001.pdf]

Supplementary Information (SI) for Nanoscale.
This journal is © The Royal Society of Chemistry 2025

Electronic Supplementary Information

Ultrasensitive Detection of 2,4-Dichlorophenoxyacetic Acid by Inhibiting Alkaline Phosphatase Immobilized onto Highly Porous Gold Nanocoral Electrode

Angelo Tricase,[a,b,§] Michele Catacchio,[a,b,§] Verdiana Marchianò,[a,b] Eleonora Macchia,[a,b,c] Paolo Bollella,*[b,d] and Luisa Torsi [b,d]

[a]*Department of Pharmacy-Pharmaceutical Sciences, University of Bari Aldo Moro, Via E. Orabona, 4 – 70125 Bari, Italy*
[b]*Centre for Colloid and Surface Science, University of Bari Aldo Moro, Via E. Orabona, 4 – 70125 Bari, Italy*
[c]*Faculty of Science and Engineering, Åbo Akademi University, 20500 Turku, Finland*
[d]*Department of Chemistry, University of Bari Aldo Moro, Via E. Orabona, 4 – 70125 Bari, Italy*
§ These authors equally contributed.

Experimental

Materials and reagents

3-morpholinopropane sulfonic acid (MOPS), 4-(2-hydroxyethyl)piperazine-1-ethanesulfonic acid (HEPES), tris(hydroxymethyl)aminomethane (TRIS), methanol (CH_3OH), ethanol (CH_3CH_2OH), ammonium chloride 99.9% (NH_4Cl), Tetrachloroauric(III) acid trihydrate 99.9% ($AuCl_3{\cdot}3H_2O$), sulfuric acid (H_2SO_4), sodium sulfate (Na_2SO_4), ascorbic acid (AA), ascorbate-2-phosphate (A2P), potassium ferrocyanide ($K_4[Fe(CN)_6]$), potassium ferricyanide ($K_3[Fe(CN)_6]$), 2,4-dichlorophenoxyacetic acid (2,4-D), magnesium sulfate ($MgSO_4$), parathion, 2,4,5-trichlorophenossyacetic acid (2,4,5-T), dichlorodiphenyltrichloroethane (DDT), phenoxyacetic acid (PA), 4-chlorophenoxyacetic acid (4-CPA) and 2-methyl-4-chlorophenoxyacetic acid (MCPA) were purchased by Merck Millipore (formerly Sigma Aldrich) and used without further purification. The enzyme orthophosphoric-monoester phosphohydrolase (AIP, EC 3.1.3.1 from Bovine Liver, 3.4 U/mg solid) was obtained by Merck Millipore (formerly Sigma Aldrich), solubilized in 10 mM HEPES buffer pH 8.5 (containing 1 mM $MgSO_4$), parceled and stored at -20 °C.

The polymeric film employed for the physicochemical enzyme immobilization was a photocross-linkable poly(vinylalcohol) with styrylpyridinium groups (PVA-SbQ) obtained from Polysciences, Inc. (USA).

All solutions for electrochemical measurements were prepared using Milli-Q water (18.2 MΩ cm, Millipore, Bedford, MA, USA).

Analysis Apparatus

All electrochemical measurements were conducted using a PalmSens4 potentiostat equipped with PSTrace 5.6v software. Commercially available screen-printed gold electrodes (DRP-220BT, named Au-SPE), comprising a circular gold working electrode (with a geometric area of 0.1256 cm^2), a gold counter electrode, and a silver pseudo-reference electrode, were purchased by Metrohm and utilized for all the electrochemical measurements (except for E-QCM). All potential values reported in the manuscript are expressed vs. Ag pseudo-reference electrode.

Scanning electron microscopy (SEM) measurements were carried out with a JSM-7600F Schottky Field Emission Scanning Electron Microscope (JEOL Nordic AB, Sollentuna, Sweden). All samples were prepared according to the electrodeposition protocol as reported above in Section 2.2 by using gold plates (25 x 25 x 1 mm, ALS Co. Ltd., Tokyo, Japan) instead of gold electrodes. The samples have been placed on a clip SEM sample holder (JEOL Nordic AB).

X-ray photoelectronic spectroscopy analyzes were carried out with Versa Probe II Scanning XPS (Physical Electronics GmbH) spectrometer and an AlKα source having a 200 µm spot. All wide-scan and high-resolution spectra were obtained in FAT mode with step energy of 117.40 eV and 29.35 eV, respectively, and with source power of 49.2 W. The charge compensation was performed with an electronic cannon operating at 1.0 V and 20.0 µA. The data were analyzed with the MultiPak v. 9.9.0.8 software.

Highly Porous Gold Nanocoral (hPGNC) Electrodeposition and Electrochemical Measurements

Au-SPE electrodes were polished via electrochemical cleaning using cyclic voltammetry in 0.5 M H_2SO_4 solution within a potential range of 0 and +1.7 for 25 cycles at a scan rate of 0.3 V s^{-1}. Afterwards, Au-SPE were modified by electrodeposition of highly porous gold nanocoral (hPGNC) by initially sweeping the potential for 25 scans between +0.8 and 0 V at a scan rate of 0.05 V s^{-1} and then applying a pulsed potential between −1 V and −3 V in 10 mM $AuCl_3$ solution containing 2.5 M NH_4Cl. [1] Then, the modified electrodes were activated in 0.5 M H_2SO_4 by running CVs between 0 and +1.7 at a scan rate of 0.1 V s^{-1} until a well-defined CV was obtained. The hPGNC

modified electrode was modified by drop-casting a solution 5:8 v/v AlP solution:PVA-SbQ (50 mg/mL) successively exposed under a UV lamp (λ = 405 nm) for 20 min at room temperature in order to allow the entrapment of the enzyme by photo-polymerization.

CVs and amperometry experiments were performed in 10 mM HEPES buffer pH 7 (containing 1 mM $MgSO_4$ as cofactor and 100 mM Na_2SO_4 as supporting electrolyte) with the addition of 2 mM AA and 2 mM A2P. The inhibition experiments are performed using 2,4-dichlorophenoxyacetic acid (2,4-D) in the range 1 fM (1×10^{-15} M) to 2 mM (2×10^{-3} M) as analytical target (acting as inhibitor of the AlP enzymatic activity) and 2,4,5-T as negative control. All measurements were performed for n=6 electrodes, each measured 3 times to increase S/N ratio.

Wheat Leaves Extract Samples Preparation

Wheat leaves were collected at 5 different local wheat farming plants harvesting the leaves in different areas of the same farm (contaminants mapping). Wheat leaves were rinsed 3-4 times with DI water to remove soil, debris, and external contaminants. The leaves were dried in a ventilated oven at 40°C until moisture content is below 10%. The dried leaves were grinded and sieved using a 60-mesh sieve for uniform particle size (~250 μm). The extraction was performed in a mixture CH_3OH:EtOH:H_2O 2:7:1 acidified with formic acid 0.1% at room temperature for 24h.

The extract was filtered with a 0.45 μm nylon filter, diluted 1:50 v/v with DI water and analysed by electrochemical methods. [2]

Results

XPS analysis

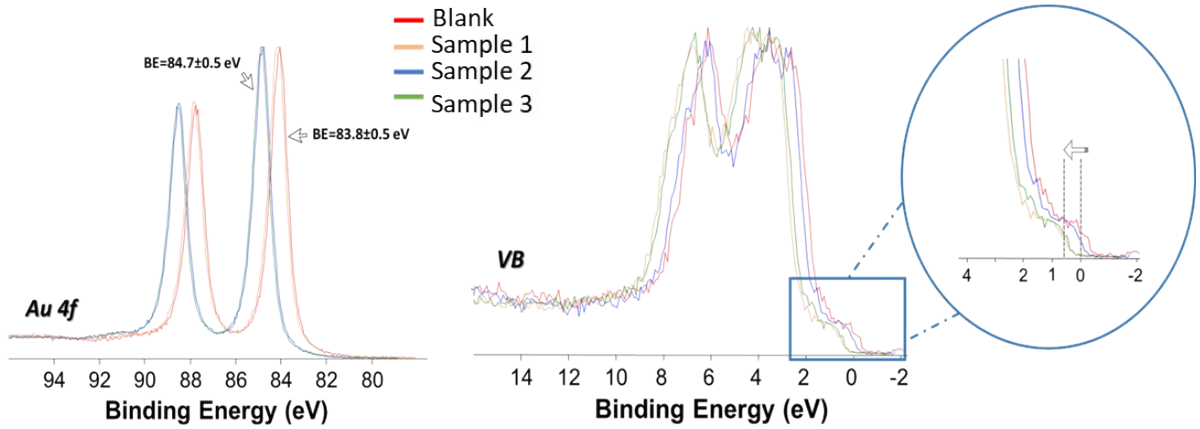

Figure S1. XP spectra of Au 4f on the left and its valence band on the right. Blank: blank (Gold bare); Sample 1: Gold SPE after cyclic voltammetry; Sample 2: Gold SPE after cyclic voltammetry and pulsed amperometry; Sample 3: Gold SPE after pulsed amperometry.

Table S1. The table shows hPGNC signals with relative chemical assignment. The values are expressed as average values ± 1σ (n = 3).

Samples	% C	% O	% Au
**Blank**	57±3	25.8±1.4	17.0±1.4
**Sample 1**	49.2±1.6	22±2	29±2
**Sample 2**	32±3	8±2	59±5
**Sample 3**	30±4	8.4±0.5	62±5

Table S2. Comparison of AlP based analytical platforms for the detection of 2,4-D. Abbreviations: alkaline phosphatase (AlP), carbon black-screen printed electrode (CB-SPE), carbon dots/cobalt oxyhydroxide nanosheet (CDs/CoOOH), gold nanobipyramids (AuNBPs), highly porous gold nanocoral (hPGNC), monoclonal antibody (mAB), paper-based analytical device (PAD), platinum (Pt), Rhodamine B modified sulfur quantum dots (RhB-SQDs).

Platform	LoD / M	Linear range / M	Storage Stability	Ref.
**AlP/AUNBs/PAD (colorimetric)**	0.08×10^{-6}	$0.2\text{-}4.5\ (\times 10^{-6})$	n.a.	[3]
**AlP/CB-SPE (electrochemical)**	0.2×10^{-6}	$0.4\text{-}2.7\ (\times 10^{-6})$	4 successive measurements	[4]
**AlP/RhB-SQDs (fluorimetric)**	0.08×10^{-6}	$0.2\text{-}2.3\ (\times 10^{-6})$	n.a.	[5]
**AlP/CDs/CoOOH (fluorimetric)**	0.5×10^{-6}	Up to 0.07	n.a.	[6]
**AlP/mAB/Pt (electrochemical)**	0.3×10^{-9}	$0.4\text{-}1500\ (\times 10^{-9})$	1 month	[7]
**PVA-SbQ/AlP/hPGNC (electrochemical)**	3.2×10^{-15}	$10\text{-}1000\ (\times 10^{-15})$	4 months	This work

References

[1] Bollella, P., Hibino, Y., Kano, K., Gorton, L. and Antiochia, R., 2018. Highly sensitive membraneless fructose biosensor based on fructose dehydrogenase immobilized onto aryl thiol modified highly porous gold electrode: characterization and application in food samples. Analytical chemistry, 90(20), pp.12131-12136.

[2] EFSA Scientific Committee. Guidance on the safety assessment of botanicals and botanical preparations intended for use as ingredients in food supplements. EFSA Journal. 2009; 7(9):1249. DOI: 10.2903/j.efsa.2009.1249.

[3] Ye, X., Zhang, F., Yang, L., Yang, W., Zhang, L. and Wang, Z., 2022. based multicolor sensor for on-site quantitative detection of 2, 4-dichlorophenoxyacetic acid based on alkaline phosphatase-mediated gold nanobipyramids growth and colorimeter-assisted method for quantifying color. Talanta, 245, 123489.

[4] Arduini, F., Cinti, S., Caratelli, V., Amendola, L., Palleschi, G. and Moscone, D., 2019. Origami multiple paper-based electrochemical biosensors for pesticide detection. Biosensors and Bioelectronics, 126, pp.346-354.

[5] Li, X., Chen, C., Xu, F., Liang, Z., Xu, G., Wei, F., Yang, J., Hu, Q., Zou, J. and Cen, Y., 2023. Novel dual-emission sulfur quantum dot sensing platform for quantitative monitoring of pesticide 2, 4-dichlorophenoxyacetic acid. Talanta, 260, 124639.

[6] Su, D., Han, X., Yan, X., Jin, R., Li, H., Kong, D., Gao, H., Liu, F., Sun, P. and Lu, G., 2020. Smartphone-assisted robust sensing platform for on-site quantitation of 2, 4-dichlorophenoxyacetic acid using red emissive carbon dots. Analytical Chemistry, 92(18), pp.12716-12724.

[7] Deng, A.P. and Yang, H., 2007. A multichannel electrochemical detector coupled with an ELISA microtiter plate for the immunoassay of 2, 4-dichlorophenoxyacetic acid. Sensors and Actuators B: Chemical, 124(1), pp.202-208.
